# The Role of Low and High Spatial Frequencies in Exogenous Attention to Biologically Salient Stimuli

**DOI:** 10.1371/journal.pone.0037082

**Published:** 2012-05-10

**Authors:** Luis Carretié, Marcos Ríos, José A. Periáñez, Dominique Kessel, Juan Álvarez-Linera

**Affiliations:** 1 Facultad de Psicología, Universidad Autónoma de Madrid, Madrid, Spain; 2 Facultad de Psicología, Universidad Nacional de Educación a Distancia, Madrid, Spain; 3 Unidad de Investigación Proyecto Alzheimer, Fundación CIEN-Reina Sofía, Madrid, Spain; 4 Facultad de Psicología, Universidad Complutense de Madrid, Madrid, Spain; 5 Departamento de Radiología, Hospital Ruber Internacional, Madrid, Spain; CNRS – Université Claude Bernard Lyon 1, France

## Abstract

Exogenous attention can be understood as an adaptive tool that permits the detection and processing of biologically salient events even when the individual is engaged in a resource-consuming task. Indirect data suggest that the spatial frequency of stimulation may be a crucial element in this process. Behavioral and neural data (both functional and structural) were analyzed for 36 participants engaged in a digit categorization task in which distracters were presented. Distracters were biologically salient or anodyne images, and had three spatial frequency formats: intact, low spatial frequencies only, and high spatial frequencies only. Behavior confirmed enhanced exogenous attention to biologically salient distracters. The activity in the right and left intraparietal sulci and the right middle frontal gyrus was associated with this behavioral pattern and was greater in response to salient than to neutral distracters, the three areas presenting strong correlations to each other. Importantly, the enhanced response of this network to biologically salient distracters with respect to neutral distracters relied on low spatial frequencies to a significantly greater extent than on high spatial frequencies. Structural analyses suggested the involvement of internal capsule, superior longitudinal fasciculus and corpus callosum in this network. Results confirm that exogenous attention is preferentially captured by biologically salient information, and suggest that the architecture and function underlying this process are low spatial frequency-biased.

## Introduction

Evolutionary success depends heavily on the efficiency of the nervous system in detecting biologically important events and reorienting processing resources to them. These stimuli tend to capture attention more efficiently than anodyne or neutral ones, both in the case of appetitive [1]–[4] and in the case of aversive distracters [5]–[7]. This efficiency relies on exogenous attention, also named automatic, stimulus-driven or bottom-up attention. Two neural circuits have been proposed as supporting exogenous attention. On one hand, the ventral attention network (VAN), comprising the temporo-parietal junction and neighboring areas in the posterior part of the superior temporal gyrus and sulcus, and the ventrolateral-caudal frontal cortex, that is, the insula and inferior/middle frontal gyri [8], [9]. This circuit would be responsible for the changeover from internally-directed processes to environmentally-directed processes [8], [10]. Certain dorsal areas (dorsal attention network –DAN-), such as the intraparietal sulcus (IPS), have been linked to dynamic representations of salience or priority maps of the environment and may serve to guide spatially targeted motor actions to cope with it [11]–[14].

By definition, events capturing exogenous attention appear outside the current focus of processing. In many situations, they also appear out of the focus of gaze, projecting to non-foveal areas of the retina and being poorly perceived until processing resources are oriented to them. Fovea covers only 1 to 2° of visual angle, roughly the area of the thumbnail at arm's length (human visual field covers 180° horizontally), so the nervous system needs to constantly assess which peripheral elements are important and deserve reorientation of controlled, limited resources to them and which do not [15]–[17]. As might be expected, this capability for processing poorly perceived events is intensified when they are important in biological terms [18]–[20]. Non-foveal processing is mainly sustained by rods, a type of retinal photoreceptor whose signals are sent to the magnocellular layers of the lateral geniculate nucleus (LGN) of the thalamus [21]. The magnocellular system conveys rudimentary visual information, poor in color and luminance details (i.e., only low spatial frequencies), but rapidly reaches “high-level" areas such as prefrontal and parietal cortices [22], [23]. On the other hand, the parvocellular system –originating in the cones, a type of photoreceptor mostly present in the fovea, and passing through the parvocellular layers of the LGN- carries precise visual information allowing deep exploration of the stimulation, but it is slower and not so extensively distributed [21].

This study explored whether exogenous attention architecture is conditioned by spatial frequency and tested the hypothesis that the observed exogenous attention network is biased to detect biologically salient stimuli. To this end, we presented background distracters projecting mainly to non-foveal areas of retina and depicting scenes of three different types – appetitive (such as food or nudes), aversive (threatening animals, aggression situations, etc.) and neutral (anodyne daily life situations and objects) – in three different spatial frequency formats (intact, low-pass filtered and high-pass filtered) while participants performed a demanding first-term cognitive task presented in foveal areas. Behavioral and brain measures, both functional (functional magnetic resonance imaging, fMRI) and structural (diffusion tensor imaging, DTI), were recorded and analyzed in a non-constrained fashion (i.e., no ROIs were defined *a priori*) to define neural areas sustaining exogenous attention to these distracters.

## Materials and Methods

### Ethics statement

The present research was approved by the Ethics Committee of the Universidad Autónoma de Madrid. All participants gave their written informed consent prior to the beginning of the experimental session described below.

### Participants

Thirty-six healthy subjects (26 women), with an age range of 19 to 38 years (mean = 24.00) voluntarily took part in this experiment (five additional subjects participated, but their recordings were eliminated due to motion [n = 3], to failure in behavioral recordings [n = 1], and to equalize the orders of presentation, as explained later [n = 1]). They gave their informed consent to participate, reporting normal or corrected-to-normal visual acuity.

### Stimuli and procedure

**Figure 1:**
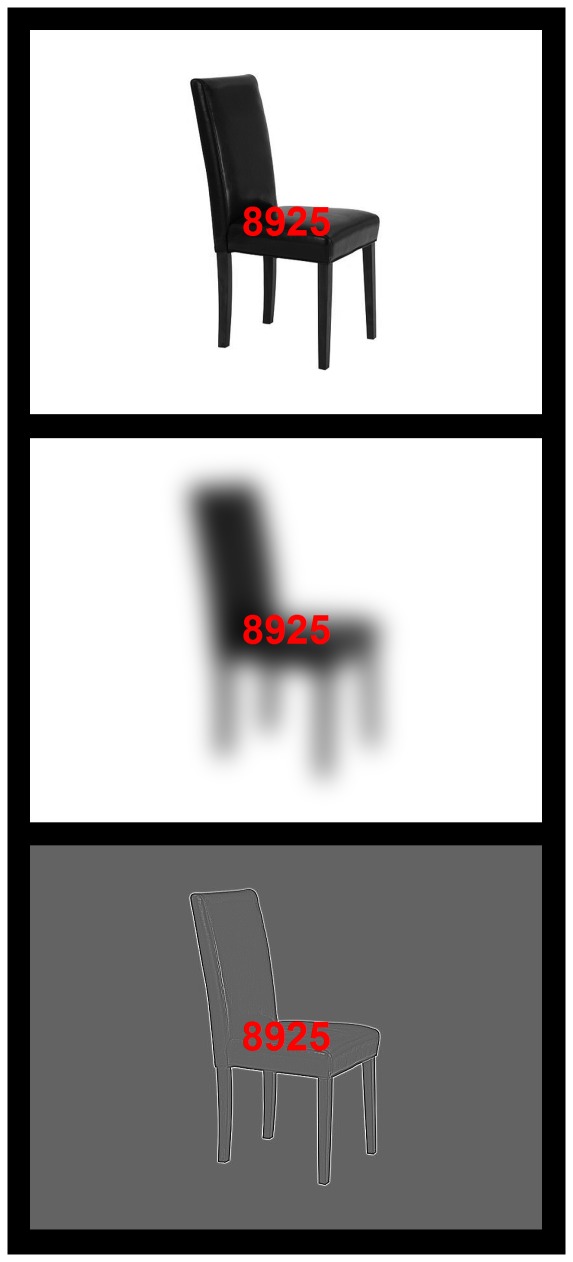
Examples of stimuli containing neutral distracters of intact, low and high spatial frequencies. For copyright reasons, original background image (distracter) has not been taken from the IAPS. Participants were asked to indicate whether the two central digits (within the string of four digits) were concordant or discordant in their even/odd condition (in the example, they are discordant).

Stimuli were presented to participants in a single run through optic-fiber-based glasses (MRVision 2000 ultra, Resonance Technology, Inc., Northridge, USA) connected to the stimulation computer. The lenses of these glasses were changeable for visual acuity correction when necessary. As illustrated in Figure 1, each presentation included a set of red digits appearing in the center of the screen (relevant to the primary task) and a black and white image in the background (distracter). Background images (30°×22.5° visual angle, i.e., mainly projected to non-foveal areas) were of nine types, which resulted from the combination of three spatial frequencies (only low or LSF, only high or HSF and intact –non filtered- or ISF) and three biological meanings (aversive/negative, neutral, and appetitive/positive), 32 of each type. All of them proceeded from 32 original aversive pictures, 32 neutral and 32 appetitive from the International Affective Picture System (IAPS [24]; see http://www.uam.es/CEACO/exoSF.htm for a list of stimuli). They were selected on the basis of their scores in valence and arousal, two affective dimensions in which IAPS pictures are assessed and which range, respectively, from unpleasant to pleasant and from calming to arousing. After the recording session, participants themselves filled out a 5-point bidimensional scale for each picture so their assessments on the valence and arousal content of the stimulation were obtained (Table 1).

**Table 1:** Means and standard error of means (in parenthesis) of: i) subjective responses to each of the three types of Biological meanings of distracters (appetitive, neutral and aversive), ii) behavioral responses (reaction times and number of errors) to each distracter type (HSF: high spatial frequency distracters; LSF: low spatial frequency distracters).

		Appetitive	Neutral	Aversive
**Sujective responses (1 to 5)**	Valence (unpleasant to pleasant)	4.171 (0.022)	3.158 (0.015)	1.714 (0.024)
	Arousal (calming to arousing)	3.595 (0.034)	2.861 (0.020)	4.129 (0.023)
**Reaction times (in milliseconds)**	HSF	874.945 (45.745)	820.181 (39.428)	844.467 (39.273)
	Intact	870.546 (34.378)	889.568 (36.054)	920.643 (45.796)
	LSF	807.702 (34.599)	831.639 (34.887)	828.955 (39.879)
**Number of errors (# trials per condition = 32)**	HSF	1.639 (0.345)	1.722 (0.375)	1.194 (0.313)
	Intact	2. 639 (0.380)	1.500 (0.294)	1.861 (0.322)
	LSF	1.722 (0.281)	1.694 (0.316)	1.861 (0.312)

Differences in spatial frequency and luminosity of intact images –from which LSF and HSF are derived- were contrasted with respect to biological meaning levels in order to discard the effect of these potential confounds in experimental effects. With respect to spatial frequency, spectral energies were computed for eight frequency bands (plus residuals) within each picture following the procedure described by Delplanque and colleagues [25] (http://www.affective-sciences.org/spatfreq), and submitted as dependent variables (one for each band) to a MANOVA (multivariate analysis of variance) with respect to factor Biological Meaning (aversive, appetitive, neutral). No significant differences were observed (p>0.25). Univariate ANOVAs were also carried out for each frequency band, differences being non-significant (p>0.05 in all cases). The effect of average luminosity of each picture, measured through Photoshop CS3 (v 10.0; Adobe Systems, San Jose, CA), was also non-significant with respect to Biological Meaning (p>0.60). Descriptive data on luminosity and spatial frequencies for all bands, as well as details on these analyses, are available at http://www.uam.es/CEACO/exoSF.htm.

In order to manipulate the spatial frequency, these original images were converted to grayscale. All images had a resolution of 72 pixels per inch and a 1024×768 pixel size. The spatial filtering was applied by using Adobe Photoshop CS3. Low-pass filtering used the application of a Gaussian blur filter with a 24.4 pixel kernel (resulting in images low-pass filtered at ≈6 cycles per image). For the high-pass filter, we used the Adobe Photoshop high-pass filter set to a radius of 1.2 pixels (resulting in images high-pass filtered at ≈30 cycles per image), followed by an adjustment of luminance and contrast. These cut frequencies, which have been previously employed (e.g., [22]), were selected in order to avoid intermediate frequencies that would be difficult to categorize as high or low.

Each picture was displayed for 500 milliseconds, and interstimulus interval (during which the screen was black with a central white cross) was 3000 milliseconds. The 32 trials within each of the nine conditions were divided into four 8-trial blocks (28 seconds each). Consequently, 36 blocks were presented to subjects. Three orders of presentation were established: the first block was ISF neutral in 1/3 of the participants, ISF aversive in another 1/3, and ISF appetitive in the remaining 1/3 of participants. After the first block, the rest of blocks were presented following semi-random criteria, in such a way that two consecutive blocks of the same biological valence or of the same spatial frequency condition were never presented.

The cognitive task concerned the central visual element, which consisted of a sequence of 4 digits (height: 1.73° visual angle): Figure 1. Those relevant for the task were the second and the third (i.e., the central digits): participants had to press, “as accurately and rapidly as possible", one key if both the second and the third digits were even or if both were odd (i.e., if they were “concordant"), and a different key if those two central digits were of different types (i.e., if they were “discordant"). Within each of the 9 conditions, half of the trials were concordant and the other half were discordant. The first and fourth digits in the sequence, or flankers, were always different from the central digits, and were included in the sequence in order to increase task difficulty and, hence, top-down attentional demand. The even/odd condition of the flankers was controlled: within each of the 9 conditions, these flanker digits were of the same even/odd category as the central digits (1st = 2nd and 4th = 3rd) in half of the trials, and of a different category (1st≠2nd and 4th≠3rd) in the other half. A training block (eight trials, four concordant and four discordant) employing intact landscapes as background was presented prior to the 36 experimental blocks.

### Analysis of behavior

With respect to behavioral data, reaction times (RTs) and number of errors were analyzed. These analyses aimed at contrasting whether the task actually enabled salient distracters to capture attention to a greater extent than neutral distracters. As indicated in the Introduction, this effect has previously been observed in response to non-manipulated (intact) distracters. In the case of RTs, outliers, defined as responses outside the inter-trial interval (3000 milliseconds) or below 200 milliseconds, were detected in order to be ignored in the analyses. Repeated-measures MANOVAs introducing RTs and number of errors as dependent variables and Spatial Frequency (low, high, intact) and Biological Meaning (aversive, neutral, appetitive) as factors were carried out. Previously, and in order to approach data distribution to normality, RTs were log transformed, and error rates (ranging from 0 to 1) were arcsine-root transformed [26]. Post-hoc pair-wise comparisons were carried out using the Bonferroni correction procedure, which addresses the problem of multiple comparisons (alpha = 0.05). The association between behavior and BOLD activity was also tested through multiple regression using the SPM8 ad-hoc tool as described below.

### fMRI scanning and analysis

The fMRI data were acquired on a 3.0T Signa HDx MR scanner (GE Healthcare, Waukesha, WI, USA) with an eight-channel head coil (GE Coils, Cleveland, OH). Head motion was minimized with a vacuum-pack system molded to fit each subject. Functional images were obtained using a T2* weighted echo-planar imaging (EPI) sequence (echo time = 33.7 ms, flip angle 90°, matrix size 128×128, field of view 24×24 cm, repetition time = 3 s). Forty contiguous axial slices (3 mm thickness) covering the whole brain were acquired. A total of 480 scans were recorded for each participant in a single session, with the first five volumes subsequently discarded to allow for T1 equilibration effects. The data were analyzed using a general linear model in SPM8 (Wellcome Department of Imaging Neuroscience, London; www.fil.ion.ucl.ac.uk/spm) implemented in MATLAB 7 (Mathworks, Inc.). Individual scans were realigned and unwarped, slice time-corrected, normalized to a standard SPM8 template based upon the Montreal Neurological Institute (MNI) reference brain [27], and spatially smoothed by a 8 mm isotropic Gaussian kernel using standard SPM methods. The voxel dimensions of each reconstructed scan were 2×2×2 mm in the x, y and z dimensions.

Population inference was made through a two stage procedure. At the first level, a subject-specific analysis was carried out where the BOLD response for each voxel and experimental condition was modeled by a boxcar waveform convolved with a canonical hemodynamic response function plus temporal and dispersion derivatives. Statistical parametric maps of the t-statistic (SPM{t}) were generated for each subject and experimental condition, and the contrast images were stored. In a second level, random effects analysis aimed at defining those areas showing a significant association with behavior. To that aim, multiple regression analyses using the SPM8 ad-hoc tool were performed introducing the activity in each voxel of the whole brain as dependent variable and both RTs and number of errors as independent variables, number of cases being equal to subjects×conditions (324). Number of errors and RTs had been previously transformed in order to approach data distribution to normality, as explained above. Next, the resulting regression map (i.e., the map of voxels in which significant behavior-BOLD signal was significant) was used as inclusive mask in a subsequent contrast exploring biologically salient>neutral significant differences (T-map). This new contrast aimed at detecting sensitivity to biological saliency among voxels underlying exogenous attention to distracters. Voxels whose parameter estimate was over the significance threshold formed ROIs that were submitted to additional analyses described next.

Activity within each ROI was computed and exported to SPSS (SPSS inc., Chicago, Il., USA) for further fine-grained statistical analyses on the interaction of biological meaning and spatial frequency of distracters. For each ROI, subject and condition, the beta value of each voxel was multiplied by its parameter estimate in the relevant>neutral contrast described above, and the results summed together. This procedure allowed the introduction of proportional weights for those voxels forming each ROI. These ROI values were submitted to repeated-measures ANOVAs in SPSS using Spatial Frequency and Biological Meaning as factors. Post-hoc comparisons to determine the significance of pairwise contrasts were carried out using the Bonferroni correction procedure (alpha = 0.05). In all cases, anatomical location of the significant activations required a transformation from MNI space (in which SPM8 solutions are provided) to Talairach space prior to introducing coordinates in the Talairach Daemon Client [28] (http://www.talairach.org/client.html) to obtain their anatomical correspondence. To this end, Matthew Brett's *mni2tal* script, implemented in Matlab, was employed [29] (http://imaging.mrc-cbu.cam.ac.uk/imaging/MniTalairach).

### DTI scanning and analysis

DTI was conducted just before the presentation of the task and the fMRI scanning. A customized DTI pulse was employed and fractional anisotropy (FA) was calculated as an indicator of white matter microstructure. Details of the whole procedure are provided in http://www.uam.es/CEACO/exoSF.htm. Group voxel-based analyses were carried out via linear regression between FA and the relevant BOLD signal found in fMRI analyses (see above) using the multiple regression tool provided by SPM8 (see Results section for details on these contrasts). This BOLD-DTI relationship has proven to be useful to define functional-structural relationships in previous studies [30]–[32]. To ensure that the observed effects were restricted to white matter regions, an inclusive white matter mask (white matter MNI template: c2avg152T1.nii) was applied.

## Results

### Experimental effects on behavior

Table 1 shows mean RTs and number of errors in the digit categorization task in each experimental condition as a function of Spatial frequency and Biological meaning of distracters. Two relevant findings were revealed by analyses on task performance. First, repeated-measures MANOVAs introducing transformed RTs and errors as dependent variables (see Materials and Methods) showed significant main effects of Spatial frequency (F(4,32) = 37.328, p<0.001, η^2^_p_ = 0.824). Bonferroni post-hoc tests signaled “natural" intact (non-filtered) and LSF distracters as those causing the greatest number of errors, and intact distracters as those eliciting the longest RTs. Second, repeated-measures ANOVAs showed significant effects of the interaction of the two factors (F(8,28) = 8.773, p<0.001, η^2^_p_ = 0.715). Interestingly, in the intact condition and as in previous experiments, the poorest performance in the ongoing cognitive task was found for biologically salient distracters: aversive in the case of RTs (which were significantly longer than those for appetitive distracters) and appetitive in the case of number of errors (which was significantly greater than for neutral and aversive distracters). When appetitive and aversive categories were collapsed into one “biologically salient" category, MANOVA showed significant main effects of Biological Meaning (F(2,34) = 3.758, p<0.05, η^2^_p_ = 0.181), salient distracters eliciting greater RTs and errors than neutral.

### Experimental effects on neural activity (fMRI)

**Figure 2:**
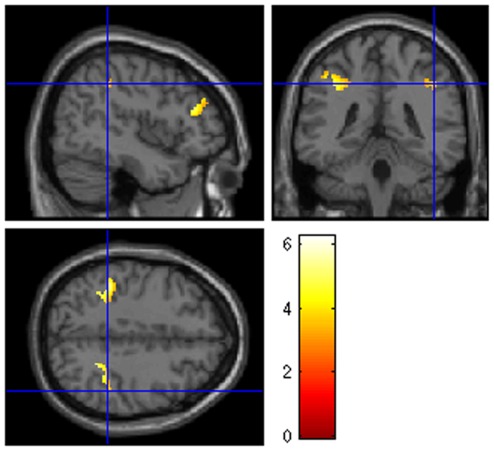
T-contrast maps showing significant biologically Salient>Neutral differences using regression map BOLD-behavior (see the main text) as inclusive mask (p<0.001, unc., cluster threshold = 30). For display purposes, statistical maps were overlaid on the Colin Holmes 27 (ch2) template of the international consortium for brain mapping (ICBM). Presented in neurological convention: R = R.

As previously described (Materials and Methods section), first step in analyses on neural activity was to detect brain areas underlying behavioral responses, which indexed exogenous attention to distracters. To that aim, a statistical map was computed involving areas showing significant associations with both normalized RTs and number of errors in multiple regression analyses carried out to that aim in SPM8 (alpha = 0.05, FWE correction). As also described, the second step was detecting, within this regression map, those areas sensitive to biological saliency of stimulation. Using the regression map as inclusive mask, T-contrasts on the relevant>neutral differences (p<0.001, uncorrected – correction was not judged as necessary since this contrast was carried out on a reduced number of voxels -, cluster threshold = 30) were carried out. As shown in Figure 2, three clusters presented greater activity to biologically salient than to neutral distracters: left and right intraparietal sulcus (rIPL-lIPL; BA7/40) and right middle frontal gyrus (rMFG; BA46, extending to the frontier with BA10). Peak voxel coordinates of each ROI are shown in Table 2. Importantly, the activity of the three ROIs strongly correlated to each other, suggesting that they responded coordinately and acted as a network (rIPL-lIPL: r = 0.715; rIPL-rMFG: r = 0.409; lIPL-rMFG: r = 0.399; bilateral p<0.001 in all cases).

**Table 2:** Clusters showing biologically Salient>Neutral significant differences (p<0.001, unc., cluster threshold = 30) after applying an inclusive mask containing voxels significantly associated with behavior (p<0.05, FWE corrected, cluster threshold = 0). Coordinates correspond to the peak voxel within each ROI. BA = Brodmann area; rIPS = right intraparietal sulcus; lIPS = left intraparietal sulcus; rMFG = right middle frontal gyrus.

ROI	x, y, z (MNI)	x, y, z (Talairach)	BA
rIPS	26, −48, 44	26,−44,43	7/40
lIPS	−32,−52,48	−32,−48,47	7/40
rMFG	42,44,34	42,44,29	46/10

**Figure 3:**
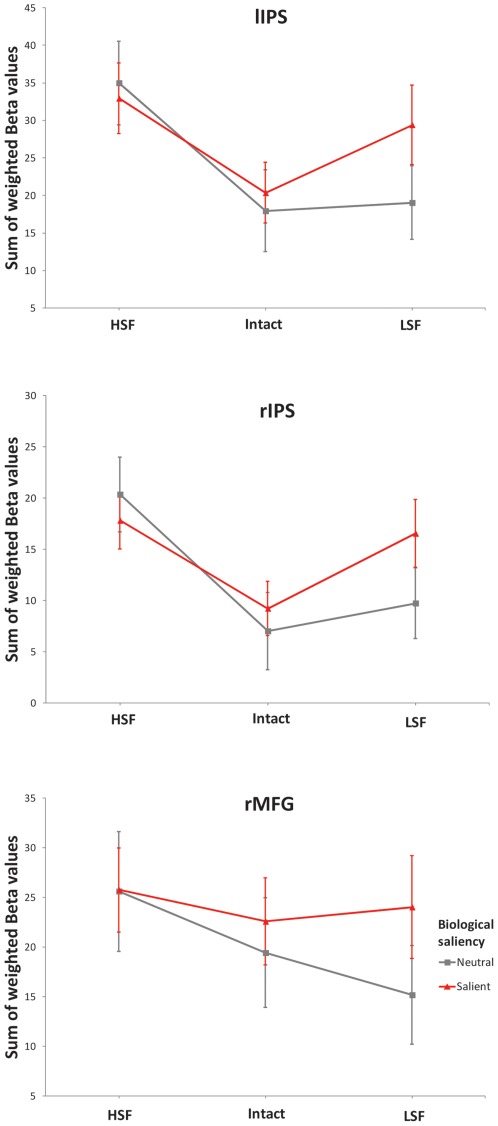
Average response of intraparietal sulci (rIPS and lIPS) and right middle frontal gyrus (rMFG) ROIs in response to Biological Saliency and Spatial Frequency. Error bars depict standard error of means. HSF = high spatial frequency distracters, LSF = low spatial frequency distracters.

The activity in these ROIs was computed through the weighted procedure explained in Procedure and Methods section (the weight of each voxel in ROI activity quantification was proportional to its parameter estimate in the salient>neutral contrast) and exported to SPSS. Taking into account that behavior showed that salient distracters (both aversive and appetitive) caused interference in the digit categorization task, these two categories were collapsed into one “biologically salient" category. Therefore, a repeated-measure ANOVA using Biological Saliency (Salient, Neutral)×Spatial Frequency (HSF, LSF)×ROI (lIPS, rIPS, BA46) was carried out. Relevant results were those related to the interaction of Spatial Frequency×Biological Saliency, which was significant (F(1,35) = 5.604, p<0.025, η^2^_p_ = 0.138). Bonferroni post-hoc tests (alpha = 0.05) showed that salient LSF distracters elicited greater activation in the three ROIs than neutral LSF distracters, while no salient versus neutral differences were observed in response to HSF distracters (Figure 3). Confirming that the three ROIs behaved as a coherent functional network, the interaction Biological Meaning×Spatial Frequency×ROI was not significant (F(2,70)<1).

Additional ANOVAs were carried out introducing intact stimuli in analyses to test their contribution to the spatial frequency effects. Since this type of distracters combined both high and low spatial frequencies, it was necessary to subtract BOLD responses to non-relevant frequencies. Thus, one ANOVA was carried out on low spatial frequencies by summing ROI responses to LSF and intact stimuli (both contained this frequency band) and subtracting responses to HSF distracters in order to discount the part of the intact distracter effects due to high spatial frequencies. Biological Saliency (Salient, Neutral) and ROI (lIPS, rIPS, BA46) were introduced as factors. Results confirmed that BOLD responses to low spatial frequencies showed salient>neutral differences (F(1,35) = 6.151, p<0.020, η^2^_p_ = 0.149). The second ANOVA was carried out on high spatial frequencies by summing ROI responses to HSF and intact distracters (both contained this frequency band) and subtracting responses to LSF stimuli. In this case, neutral>salient differences were observed (F(1,35) = 10.389, p<0.005, η^2^_p_ = 0.229).

### Structural analyses (DTI)

**Figure 4:**
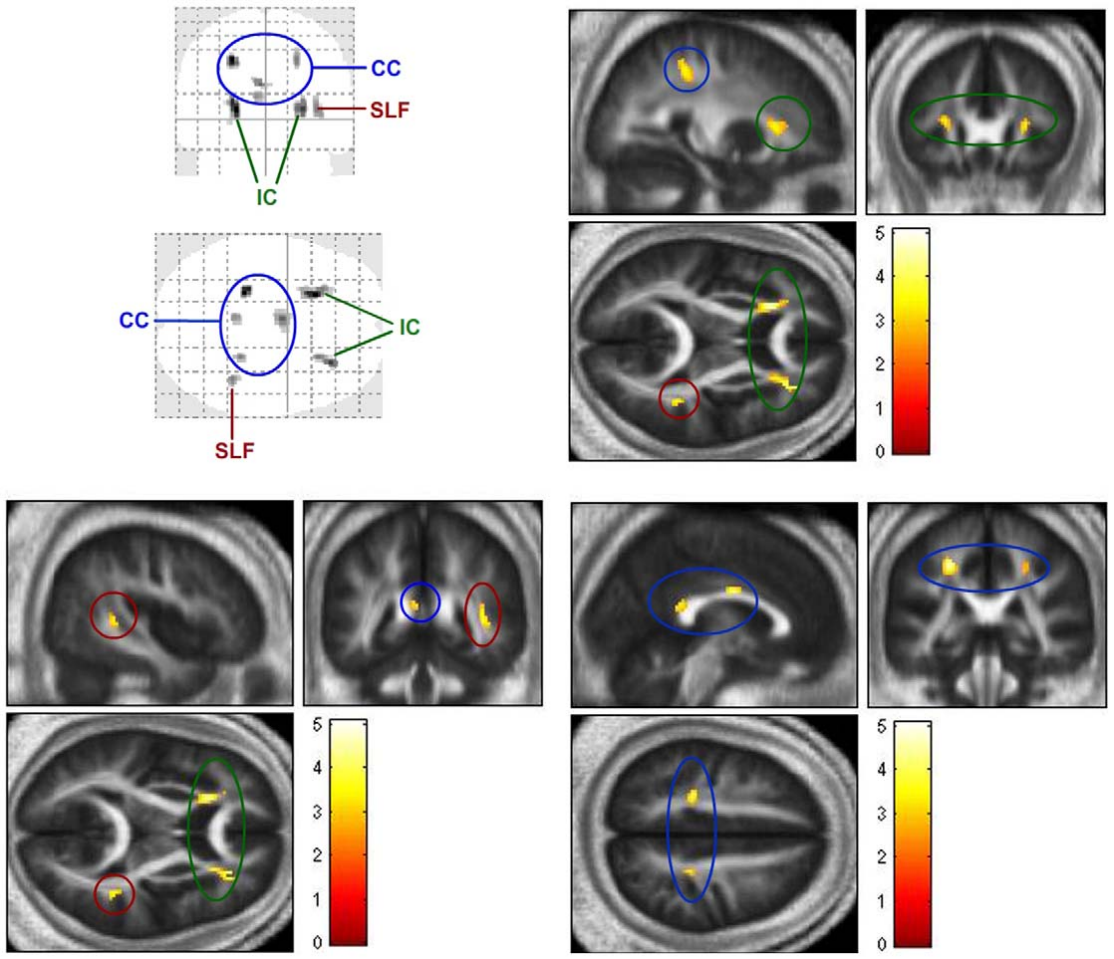
White matter clusters in which FA showed a significant positive association with BOLD activity at intraparietal sulci (rIPS and lIPS) and middle frontal gyrus (MFG) ROIs (**Figure 3**) (p<0.01, unc., cluster threshold = 30). Statistical maps are overlaid on the average of normalized FA images from the n = 36 sample itself (presented in neurological convention: R = R). IC = internal capsules (green), SLF = superior longitudinal fasciculus (red), CC = corpus callosum (blue).

Structural analyses tested whether white matter microstructural characteristics could be related to the activity observed in the LSF circuit defined above. To that aim, two multiple linear regression analyses were computed in SPM 8 between FA at every voxel within whole brain's white matter (dependent variable) and the weighted BOLD signal at lIPS, rIPS and rMFG ROIs (three separate independent variables). Thus, no ROI restrictions, except the white matter mask of the whole brain (specified in Materials and Methods section), were introduced. In the first analysis, BOLD signals introduced in the model were those recorded in response to LSF distracters. As shown in Figure 4, this structural-functional correlation was significant in three white matter areas (alpha = 0.01, unc., cluster threshold = 30): the anterior limb of bilateral internal capsules, right superior longitudinal fasciculus –subcomponent II/III-, and body and splenium of corpus callosum. Interestingly, in the second regression analysis, in which BOLD activity introduced in the model was that elicited by HSF distracters, no significant associations were observed at any voxel between FA and IIPS, rIPS and MFG activity using the same statistical constraints (alpha = 0.01 unc., cluster threshold = 30).

## Discussion

As expected, biologically salient distracters were associated with an enhanced behavioral pattern of exogenous attention. Thus, they caused poorer execution in the digit categorization task than neutral distracters, signaling a greater capability to capture attention. This behavioral pattern was associated with a neural network which appears to mainly rely on the information conveyed by low spatial frequencies contained in stimuli capturing attention. Next, this neural network will be described and discussed from both a functional and a structural perspective. Common conclusions and several implications of the results are presented at the end of this section.

### Function: IPS – MFG

The activity of left and right IPS (BA 7/40) and right MFG (BA46) was significantly associated with behavioral indices of exogenous attention to distracters. Response of the three clusters was strongly correlated to each other, so they can be understood as a coherent functional network in response to the experimental conditions employed in this study. This network showed enhanced activity to biologically salient distracters when they presented only low spatial frequencies, but not when they included only high spatial frequencies.

The involvement of IPS in attention, both exogenous and endogenous, is a consistent finding (see a review in [8]). Interestingly, TMS applied to this area reduces attentional capture by visual distracters in target-distracter competition paradigms [33]. Functionally, IPS is located within DAN [8] and, as mentioned in the Introduction, its role (and that of surrounding areas of the posterior parietal cortex) has been linked to the location or mapping of the different elements perceived in our environment according to their importance [11]–[14]. This ‘salience map’ would locate each element in the visual scene with respect to the current orientation of the body and the head, a necessary step to organize ocular and limb behavior. Indeed, IPS is involved in gaze control [34], [35]. Present results suggest that the onset of biologically salient stimuli causes IPS to actualize the priority map when a reorientation of processing resources is required.

Middle frontal gyrus (MFG) has been proposed to belong to both VAN and DAN, being the point in which both networks link together [8]. Supporting the idea that MFG is involved in DAN, present results showed that activity of rMFG strongly correlated with that of left and right IPS. A more immediate and specific functional interpretation may be provided for the activity of this prefrontal region in our study. Activity of MFG is observed in covert rather than overt shifts of attention (see a meta-analysis in [36]). Indeed, models of ocular control attribute MFG a crucial role in saccade inhibition [35]. The ability of the antisaccade task to suppress reflexive saccades towards the visual target critically depends on the integrity of BA 46 specifically [37], the area which was activated in the present experiment. The task employed here asked subjects to fixate their gaze in a central cross, and to avoid any ocular movement. Although the possibility that participants sporadically moved their eyes to explore pictures serving as distracters (and hence, the possibility that attention shifts were not only covert but also overt) may not be discarded since ocular movements were not recorded, short exposure times and difficulty of the task, along with explicit instructions given to participants, would have favored fixation of gaze in the central digits and the inhibition of saccades. In any case, further research measuring the actual locus of gaze is necessary to confirm current results and to control the effect of other, overt and non exogenous, attentional processes.

### Structure: internal capsule, superior longitudinal fasciculus and corpus callosum

This experiment also aimed at exploring the structural circuitry underlying the functional network described above. Three white matter pathways were revealed as important at this respect: the anterior limb of left and right internal capsules, the right superior longitudinal fasciculus (SLF II/III), and the corpus callosum (body and splenium). Their fractional anisotropy was positively associated with BOLD signal of rIPS, lIPS and rMFG in response to LSF distracters, but not to HSF distracters. Internal capsule (usually divided into anterior, genu and posterior parts) contains afferent pathways to neocortex as well as corticoefferent pathways [38]. Recent studies correlating structural and functional measures, as the present one, have reported the involvement of the anterior limb of internal capsule in attentional processes. For example, its white matter microstructure correlates with alert-related components of attention [39] and with attention shifting capabilities [40]. In relation to this, it is important to indicate that parietal and dorsal frontal control of eye movements is carried out through cortico-collicular efferences that travel through the internal capsule [35], [41]. Particularly, BA46 region –responsible, as indicated, of inhibiting saccades- projects through the anterior limb of the internal capsule to the superior colliculus (SC; see a review in [42]), a mesencephalic structure is part of a network of brain areas that control gaze. However, it also contributes to the control of covert spatial attention: different regions of the visual field receive enhanced attention, even in the absence of ocular movements, as a function of the collicular area being stimulated [43]. As the SC is a small subcortical structure, the volume of activation in this area was probably too small to surpass statistical constraints introduced in the present study (see [44] for a review of studies on spatial attention failing to detect superior colliculus activation for similar reasons).

Additionally, structural analyses showed a positive relationship between BOLD responses to low frequency conditions and FA in both the superior longitudinal fasciculus (subcomponent II or III) and the corpus callosum (body and splenium). The superior longitudinal fasciculus tract interconnects dorsal prefrontal cortex and parietal cortex [45], including areas in charge of controlling gaze [34]. Although the superior longitudinal fasciculus has traditionally been associated with preparation for action (particularly the subcomponent I: [45]), its involvement in visual attention, and particularly in the exogenous capture of attention by relevant events, has recently been reported [46]. Corpus callosum is the main pathway of communication between both hemispheres, and intervenes whenever the information from the two visual hemifields must be taken into account for motor behavior [47], as is the case in the digit categorization task employed in this study. Besides this “low" or visuomotor level, corpus callosum intervenes in interhemispheric communication during complex, “higher" cognitive processes that involve circuits distributed in both hemispheres [48]. Present results suggest that low-frequency dependent exogenous attention is among these processes.

### Conclusions and implications

Results reveal that at least part of the exogenous attention circuitry is biased towards biologically salient stimuli and mainly relies on low spatial frequencies to accomplish its scope of redirecting processing resources towards the attention capturing event. Both conclusions are important in evolutionary terms, and are interrelated. Thus, the preeminence of low spatial frequencies in the IPS/MFG network suggests that these structures respond mainly to magnocellular information. Indeed, IPS is set at the end of the dorsal visual stream, in which magnocellular activation is the dominant signal (see a review in [49]). Both IPS and MFG may receive this magnocellular information from early visual areas or directly from the thalamus, and in any case they are characterized by an extremely rapid response capability (<80 milliseconds in non-human primates: [50]). The idea of a magnocellular-based architecture of exogenous attention, and its associated processing speed, seems reasonable taking into account that biologically salient stimuli often require urgent responses. In relation to this, and in order to complement present data, the temporal dynamics of the observed activity are worth to be explored through agile neural signals in future research.
