# Correction: The Role of Low and High Spatial Frequencies in Exogenous Attention to Biologically Salient Stimuli

**DOI:** 10.1371/annotation/e7488e7f-1c67-4718-8afc-604b4214d409

**Published:** 2012-05-24

**Authors:** Luis Carretié, Marcos Ríos, José A. Periáñez, Dominique Kessel, Juan Álvarez-Linera

There was an error in the published funding statement. The correct funding is: This work was supported by the grants PSI2008-03688 and PSI2011-26314 from the Ministerio de Economía y Competitividad (MINECO) of Spain (http://www.mineco.gob.es/stfls/mineco/investigacion.html<http://www.mineco.gob.es/stfls/mineco/investigacion.html> [^] ). The funders had no role in study design, data collection and analysis, decision to publish, or preparation of the manuscript. 

